# Interaction between antibiotic use and MS4A2 gene polymorphism on childhood eczema: a prospective birth cohort study

**DOI:** 10.1186/s12887-021-02786-x

**Published:** 2021-07-14

**Authors:** Li Hua, Qian Chen, Quan-Hua Liu, Yi-Feng Guo, Ru-Hong Cheng, Jun Zhang, Jian-Hua Zhang, Li-Wei Wang, Ruo-Xu Ji

**Affiliations:** 1 Department of Pediatric Pulmonology, Xin Hua Hospital, Shanghai Jiao Tong University School of Medicine, 1665 Kongjiang Road, Shanghai, 200092 China; 2 Ministry of Education-Shanghai Key Laboratory of Children’s Environmental Health, Xin Hua Hospital, Shanghai Jiao Tong University School of Medicine, 1665 Kongjiang Road, Shanghai, 200092 China; 3 Department of Dermatology, Xin Hua Hospital, Shanghai Jiao Tong University School of Medicine, 1665 Kongjiang Road, Shanghai, 200092 China

**Keywords:** Eczema, Antibiotic use, Gene, *MS4A2*, Birth cohort

## Abstract

**Background:**

Eczema is usually the first allergic manifestation to appear in life attributed to gene–environment interactions. *IL13*, *IL4*, *MS4A2* and *ILR4A* are four key inflammatory genes associated with atopy. This study aimed to explore gene-environment interactions on eczema in early life among the above four genes and environmental factors in Chinese Han children.

**Methods:**

Five hundred ninety-seven children from a birth cohort who completed two-year follow-up were enrolled and their cord blood was collected. Subjects were genotyped for six polymorphisms in the aforementioned four genes. The children were followed at 6, 12 and 24 months, with epidemiologic information and medical history of eczema collected by questionnaire and eczema assessed by dermatologists.

**Results:**

Among the 597 children, 168 were diagnosed with eczema and the others were not after 2 years of follow-up. *MS4A2* rs569108 GG genotype (*P* = 1.68E-02, odds ratio (OR) = 4.66) and antibiotic use (*P* = 3.75E-4, OR = 2.02) were found independently associated with development of childhood eczema. Children with both antibiotic use and *MS4A2* rs569108 GG genotype were more likely to develop eczema than those with only antibiotic use or GG homozygote (OR = 6.24 VS. 2.04 or 4.68).

**Conclusions:**

*MS4A2* rs569108 polymorphism and antibiotic use were solely associated with eczema, and they interacted with each other to increase the risk of developing the disease in Chinese Han toddlers. Long-term follow-up along with functional and replication studies are still needed.

## Introduction

Eczema, also known as atopic dermatitis, is a common inflammatory skin disease beginning in early life with increasing incidence in many countries around the world [1, 2]. The high prevalence of eczema is believed to be a result of gene-environment interactions [3, 4], which has aroused extensive research interest and led to increasing studies on the risk and protective factors of the disease.

Allergic diseases have long been attributed to IgE-mediated inflammatory reactions [5] and eczema is usually the first allergic manifestation to appear in life [6]. In this study, we focused on four key inflammatory genes affecting IgE levels, including *IL13*, *IL4*, *MS4A2* and *ILR4A*, which have been associated with atopy [7–12], and replicated in more than ten different studies [13]. We attempted to explore gene-environment interactions on eczema in early life among the aforementioned four genes and environmental factors in a birth cohort with a two-year follow-up in Chinese Han population.

## Methods

### Study design and participants

A birth cohort study was carried out at two large tertiary hospitals in Shanghai from 2012 to 2015. One thousand fifty-six women who had a singleton pregnancy and planned to live in Shanghai for at least 2 years were recruited. Trained research nurses conducted face-to-face interviews to collect parental information on atopy, education levels and family income. Newborn’s birth information from hospital records along with umbilical cord blood were collected at birth by research nurses. Then the children were followed up for 2 years and their medical history of eczema and epidemiologic information were collected by questionnaire. Five hundred ninety-seven Chinese Han children who completed the 2 years of follow-up were included in this study. Written informed consent was obtained from parents and / or legal guardians for all the subjects who are under 16. This study was approved by the Ethics Committees of the two participating hospitals, Xinhua Hospital and the International Peace Maternity & Child Health Hospital, and conducted according to the principles in the Declaration of Helsinki.

### Questionnaire survey and assessment of eczema

Each study subject was followed up at 6 months, 1 year and 2 years of age. At six-month follow-up, an internet-based questionnaire survey was conducted to collect children’s history of eczema via the standardized International Study of Asthma and Allergies in Childhood (ISAAC) questionnaire, which had been validated and adapted in China [14, 15]. Information on antibiotic use and environmental exposures, including home pet sitting, secondhand smoke exposure and home decoration was also collected. At one-year and two-year follow-up visits, face-to-face questionnaire interviews were conducted to collect similar information as that at six-month follow-up.

The diagnostic criteria for eczema were: itchy rash on the flexural sites (the folds of the elbows, behind the knees or in front of the ankles), face, or around the neck or ears, and itchy rash coming and going for at least 6 months, based on the UK working party’s diagnostic criteria [16]. Two dermatologists diagnosed eczema independently for all the subjects based on the questionnaires, with disagreements resolved by consensus.

### Candidate genes and single nucleotide polymorphisms

This study focused on four candidate genes, including *IL13*, *IL4*, *MS4A2* and *IL4RA*, which are key inflammatory genes associated with IgE levels [7–12]. Within these genes, six known functional single-nucleotide polymorphisms (SNPs) with minor allele frequency greater than 10% based on Chinese population [17] were chosen for analysis, as shown in Table 1.

**Table 1:** Candidate genes and SNPs analyzed in this study

Gene	SNP	Chromosome position	Location	Allele ^a^	MAF ^b^
*IL13*	rs20541	5:132660272	Exon 4	G/A	0.34/0.20/0.20
*IL4*	rs2243250	5:132673462	Promoter	T/C	0.21/T, 0.15/0.36 ^c^
*MS4A2*	rs1441586	11:60088555	Promoter	T/C	0.35/0.44/0.49
*MS4A2*	rs569108	11:60095631	Exon7	A/G	0.18/0.03/0.17
*IL4RA*	rs1805010	16:27344882	Exon 5	A/G	0.50/0.45/0.48
*IL4RA*	rs1801275	16:27363079	Exon 12	A/G	0.18/0.20/A, 0.34 ^d^

*SNP* single-nucleotide polymorphism, *MAF* minor allele frequency

^a^ Major/minor allele shown

^b^ MAF in this study/Europeans/Africans. MAF in Europeans and Africans was from National Center for Biotechnology Information (https://www.ncbi.nlm.nih.gov/)

^c^ Minor allele is C in this study and Africans, but it is T in Europeans

^d^ Minor allele is G in this study and Europeans, but it is A in Africans

### Genotyping

Genomic DNA was obtained from umbilical cord blood with the QIAamp DNA Blood Mini Kit (Qiagen, Hilden, Germany). SNP genotyping was performed using matrix-assisted laser desorption / ionization time of flight mass spectrometry (MALDI-TOF MS) [18] (MassArray iPLEX; Sequenom, San Diego, CA, USA). Genotyping call rate for each SNP exceeded 99%. 5% of the samples were blindly retested and concordance rate for duplicate genotyping was greater than 98%.

### Statistical analysis

Associations of epidemiologic factors with eczema were assessed by multivariate logistic regression adjusting for potential confounders. Each SNP was tested for Hardy–Weinberg equilibrium in the study subjects with the χ^2^ test, and associations of the SNPs with eczema were also evaluated using multivariate logistic regression. Gene - environment interaction on eczema was examined by χ^2^ tests. A two-tailed *P* value of 0.05 or less was considered statistically significant. All analyses were performed using SPSS version 17.0 (IBM Corp., Armonk, NY, USA).

## Results

### Associations of epidemiologic factors with eczema

Among the 597 children, 168 were diagnosed with eczema after 2 years of follow-up while the others were not. Characteristics of the study subjects were presented in Table 2. Antibiotic use was found significantly associated with development of childhood eczema after adjusting for multiple child and parental characteristics (*P* = 3.75E-4, odds ratio (OR) = 2.02). However, baby’s gender, gestational age, birth weight, delivery mode, parity, home pet sitting, in-home secondhand smoke exposure, home decoration, family income, parental atopy and education level were not found associated with eczema by multivariate logistic regression (*P* > 0.05).

**Table 2:** Associations of epidemiologic factors with eczema

Phenotypes	Eczema n (%)	Non-eczema n (%)	*P* value ^a^	OR (95% CI) ^b^
Gender				
Boy	95 (56.9)	212 (49.4)		1
Girl	72 (43.1)	217 (50.6)	0.20	0.78 (0.53, 1.14)
Gestational age (wk)				
< 37	3 (1.8)	18 (4.2)		1
37–39	126 (75.0)	293 (68.3)	0.44	1.84 (0.39, 8.61)
≥ 40	39 (23.2)	118 (27.5)	0.64	1.47 (0.30, 7.15)
Birth weight (g)				
< 2500	2 (1.2)	14 (3.3)		1
2500–4000	152 (90.5)	377 (87.9)	0.44	2.07 (0.32, 13.24)
≥ 4000	14 (8.3)	38 (8.9)	0.48	2.04 (0.28, 14.68)
Delivery mode				
Vaginal	44 (26.2)	105 (24.5)		1
Cesarean section	124 (73.8)	324 (75.5)	0.31	0.80 (0.51, 1.23)
Parity				
None	158 (94.0)	390 (90.9)		1
≥ 1	10 (6.0)	39 (9.1)	0.16	0.57 (0.26, 1.25)
Antibiotic use				
No	72 (42.9)	255 (60.0)		1
Yes	96 (57.1)	170 (40.0)	0.00	2.02 (1.37, 2.98)
Home pet sitting ^c^				
No	133 (79.2)	355 (83.5)		1
Yes	35 (20.8)	70 (16.5)	0.51	1.18 (0.72, 1.92)
In-home secondhand smoke exposure				
No	66 (39.3)	213 (50.2)		1
Yes	102 (60.7)	211 (49.8)	0.16	1.32 (0.89, 1.96)
Home decoration				
No	152 (91.0)	394 (92.7)		1
Yes	15 (9.0)	31 (7.3)	0.43	1.31 (0.67, 2.57)
Parental atopy ^d^				
No	120 (72.3)	346 (81.6)		1
Yes	46 (27.7)	78 (18.4)	0.06	1.53 (0.98, 2.39)
Maternal education				
Middle school or lower	3 (1.8)	15 (3.5)		1
High school	14 (8.3)	57 (13.3)	0.87	1.13 (0.24, 5.40)
College or higher	151 (89.9)	356 (83.2)	0.43	1.81 (0.42, 7.85)
Paternal education				
Middle school or lower	2 (1.2)	6 (1.4)		1
High school	17 (10.2)	51 (12.0)	0.65	0.64 (0.09, 4.45)
College or higher	148 (88.6)	367 (86.6)	0.44	0.48 (0.07, 3.11)
Family income (CNY)				
< 100 K	44 (26.3)	129 (30.2)		1
≥ 100 K	101 (60.5)	226 (52.9)	0.24	1.32 (0.84, 2.08)
Unknown	22 (13.2)	72 (16.9)	0.77	0.91 (0.49, 1.70)

*OR* odds ratio, *CI* confidence interval

^a^ *P* Values were tested by multivariate logistic regression

^b^ All parameter estimates were adjusted for other covariates

^c^ keeping cats or dogs at home

^d^ Parental atopy was referred to those parents who had asthma, allergic rhinitis or atopic dermatitis along with detectable specific IgE

### Associations of candidate genes with eczema

All the SNPs met Hardy-Weinberg equilibrium criteria (*p* > 0.05). The genetic models (additive, dominant and recessive models) were tested for the SNPs. Among the six SNPs, only MS4A2 rs569108 was found associated with childhood eczema (P < 0.05), and its most significant association with the disease was under additive model. Association test results under additive model were shown in Table 3. *MS4A2* rs569108 GG genotype was found significantly associated with eczema (*P* = 1.68E-02, OR = 4.66). No significant associations were found between the other five SNPs and eczema (*P* > 0.05).

**Table 3:** Associations of candidate genes with eczema

Genotypes	Eczema n (%)	Non-eczema n (%)	*P* value ^a^	OR (95% CI)
*IL13* rs20541				
AA	13 (7.7)	52 (12.1)		1
AG	79 (47.0)	197 (46.0)	0.13	1.70 (0.86, 3.36)
GG	76 (45.2)	179 (41.8)	0.10	1.77 (0.90, 3.49)
*IL4* rs2243250				
CC	8 (4.8)	21 (4.9)		1
CT	55 (32.7)	137 (31.9)	0.73	1.17 (0.48, 2.88)
TT	105 (62.5)	271 (63.2)	0.83	1.10 (0.46, 2.62)
*MS4A2* rs1441586				
CC	20 (11.9)	46 (10.8)		1
CT	70 (41.7)	212 (49.8)	0.73	1.14 (0.53, 2.46)
TT	78 (46.4)	168 (39.4)	0.41	1.42 (0.61, 3.30)
*MS4A2* rs569108				
AA	120 (71.4)	285 (66.4)		1
AG	38 (22.6)	137 (31.9)	0.26	0.74 (0.44, 1.25)
GG	10 (6.0)	7 (1.6)	0.02	4.66 (1.32, 16.44)
*IL4RA* rs1805010				
AA	44 (26.3)	109 (25.6)		1
AG	79 (47.3)	209 (49.1)	0.82	0.95 (0.61, 1.49)
GG	44 (26.3)	108 (25.4)	0.67	1.12 (0.67, 1.88)
*IL4RA* rs1801275				
AA	107 (63.7)	288 (67.3)		1
AG	59 (35.1)	127 (29.7)	0.10	1.41 (0.94, 2.10)
GG	2 (1.2)	13 (3.0)	0.25	0.41 (0.09, 1.86)

*OR* odds ratio, *CI* confidence interval

^a^ *P* Values were tested by multivariate logistic regression

### Interaction between antibiotic use and *MS4A2* rs569108 on eczema

Table 4 shows that children with both antibiotic use and *MS4A2* rs569108 GG genotype were more likely to develop eczema than those without antibiotic use and GG homozygote (*P* = 1.49E-02, OR = 6.24), and also more likely to develop eczema than those with only antibiotic use or GG genotype (OR = 6.24 VS. 2.04 or 4.68).

**Table 4:** Interaction between antibiotic use and *MS4A2* rs569108 on eczema

Antibiotic use	rs569108	Eczema n (%)	Non-eczema n (%)	*P* value ^a^	OR(95%CI)
–	AA/AG	67 (39.9%)	251 (59.1%)		1
–	GG	5 (3.0%)	4 (0.9%)	0.03	4.68 (1.22,17.92)
+	AA/AG	91 (54.2%)	167 (39.3%)	0.00	2.04 (1.41,2.96)
+	GG	5 (3.0%)	3 (0.7%)	0.01	6.24 (1.46,26.79)

*OR* odds ratio, *CI* confidence interval

^a^ *P* Values for χ^2^ tests

## Discussion

In this study, *MS4A2* rs569108 GG genotype and antibiotic use were found solely associated with eczema in early life, and they interacted with each other to enhance the risk of developing the disease. This is the first report of gene-environment interactions between rs569108 and antibiotic use on childhood eczema.

Our study is the first to report an independent association of *MS4A2* rs569108 GG homozygote with eczema in two-year-old children. This polymorphism has been reported significantly associated with histamine release from basophils [19], which may induce atopic eczema [20]. Our study also confirmed the independent effect of antibiotic use on risk of childhood eczema, consistent with previous reports [21–24]. Early life exposure to antibiotics may have adverse effects on the neonatal gut microbiome and adversely affect the development of the infant immune system, leading to childhood atopic diseases [25]. How the polymorphism in the *MS4A2* gene interacts with antibiotic exposure in early life to increase risk of eczema remains unknown. Future studies are needed to investigate the biological interactions between them.

There are some limitations in this research. First, only 597 children completed two-year follow-up in this study, which had a small sample size and a short follow-up duration. Larger sample size and long-term follow-up are needed in future. Second, only four genes (i.e. *IL13, IL4, MS4A2* and *IL4RA*) were chosen as candidate genes. However, the four genes are susceptible genes of atopy [7–12] replicated in more than ten different populations [13]. In our further study, more genes and SNPs associated with atopy and eczema should be included. Third, environmental exposures were evaluated by self-reported questionnaire, which may underestimate the associations of certain environmental exposures. Direct measurement of certain environmental exposures is needed in future. Fourth, assessment of eczema was based on parental-reported symptoms in this birth cohort study. 28.14% (168/597) of the subjects developed eczema, and the prevalence data was similar with that reported in another study [26]. Fifth, replications of the findings in other populations are needed in future studies.

## Conclusions

This study suggests that *MS4A2* rs569108 polymorphism and antibiotic use were independently associated with eczema, and they interacted with each other to increase the risk of developing the disease in Chinese Han toddlers, which still need long-term follow-up along with functional and replication studies.

## Data Availability

The datasets generated and analyzed during the current study are not publicly available due to ethical concerns, but are available from the corresponding authors on reasonable request.

## Abbreviations

SNPs: Single-nucleotide polymorphisms
OR: Odds ratio
rs: Reference SNP
CI: Confidence interval
